# Synthesis and Preliminary Biological Evaluations of 5′-Substituted Derivatives of Uridine as Glycosyltransferase Inhibitors

**DOI:** 10.3390/molecules18078018

**Published:** 2013-07-08

**Authors:** Jadwiga Paszkowska, Katarzyna Kral, Tadeusz Bieg, Urszula Nawrot, Wiesław Szeja, Ilona Wandzik

**Affiliations:** 1 Department of Organic Chemistry, Bioorganic Chemistry and Biotechnology, Silesian University of Technology, B. Krzywoustego 4, 44-100 Gliwice, Poland; E-Mails: jadwiga.paszkowska@polsl.pl (J.P.); katarzyna.kral@polsl.pl (K.K.); jtadeusz.bieg@polsl.pl (T.B.); wieslaw.szeja@polsl.pl (W.S.); 2 Department of Microbiology, Wroclaw Medical University, T. Chałubińskiego 4, 50-368 Wrocław, Poland; E-Mail: urszula.nawrot@umed.wroc.pl

**Keywords:** uridine, glycosyltransferase inhibitors, antifungal activity

## Abstract

New derivatives of uridine which contain a β-ketoenol motif were synthesized, characterized and biologically tested. Synthesized compounds **1**–**4** showed no activity against bovine milk β-1,4-galactosyltransferase I at concentrations up to 2.0 mM and were not active against *Candida albicans* and *Aspergilus fumigatus* up to the maximum tested concentration of 1,000 µg/mL.

## 1. Introduction

Uridine is a structural element of many bioactive compounds. The majority of glycosyltransferases (GTs) utilise donors containing a uridine pyrophosphate leaving group (UDP). GTs are involved in several metabolic pathways and modulation of their activities by efficient inhibitors has potential for the control of certain cellular functions [1]. Enzymes belonging to the GT-A superfamily are metal- dependent enzymes which require a divalent cation, typically Mn^2+^, for their activity [2]. The metal ion is essential for catalysis since it interacts with the pyrophosphate group of the UDP-sugar donor in the enzyme active site. Different strategies have been used in order to design potent inhibitors of GTs as donor substrate analogues [3,4,5,6]. A variety of pyrophosphate analogues that are capable of mimicking pyrophosphate-metal interactions have been developed. A common approach is the substitution of a phosphoester oxygen atom by a carbon or sulphur atom, resulting in higher stability towards enzymatic hydrolysis [7,8,9]. Derivatives containing a diphosphate group or its analogues were good inhibitors, likely due to coordinated interaction. However these inhibitors are charged and therefore difficult to transport across the cell membrane. In order to improve the bioavailability compounds containing mimetics of the diphosphate group were synthesized as GTs inhibitors, e.g., malonic and tartaric acid derivatives [10], analogues containing monosaccharide moieties acting as pyrophosphate-metal ion complexes [10,11,12] or sugar-aminoacid-nucleosides [13].

The identification of new, uncharged substitutes for the diphosphate group with the ability to coordinate to a divalent metal is an important criterion for the development of potent GTs inhibitors as sugar-nucleotide mimics. The interaction of the inhibitors with the divalent metal ions in the active site of the enzyme is considered as a key factor in the inhibition of GTs. Ketoenols are thought to be involved in metal chelation and this structural fragment is very common for known inhibitors of metal dependent enzymes, e.g., HIV integrase [14]. β-Ketoenol ligands are bidentate and can bind metals via chelation, forming stable six-membered rings in metal-ligand structures. According to our best knowledge there are no reports on application of this motif in GT inhibitors.

**Figure 1:**
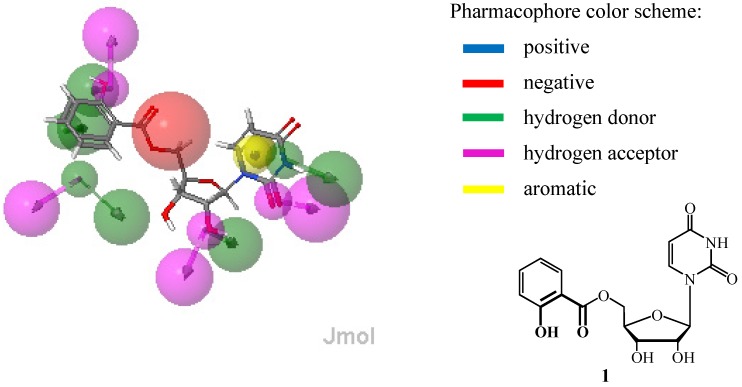
Pharmacophore of β4GalT I (PDB ID 1TVY) and aligned ligand **1** pose.

We have designed structures for the synthesis and identified potential targets using a reverse pharmacophore mapping approach with the aid of PharmMapper Server [15]. It was very promising when β-1,4-galactosyltransferase I (β4GalT I) was identified as the best target candidate from over 7,000 receptor-based pharmacophore models for molecule **1** (Figure 1). To validate the prediction we have made an effort to synthesize and test compound **1** and its derivatives as potential inhibitors of β4GalT I.

## 2. Results and Discussion

**Figure 2:**
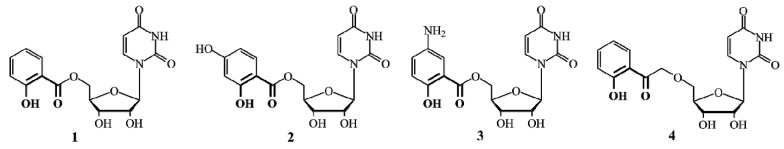
C-5′ modified uridine derivatives **1**–**4**.

In our current study we report on the synthesis of C-5′ modified uridine derivatives, which contain β-ketoenol motif (Figure 2). The two functional groups of this motif: hydroxyl and carbonyl are connected with the uridine part by either an ester or ether linkage. The uridine part is treated as a necessary moiety for the recognition and proper binding in the enzyme active site. Differential benzene ring substitution was introduced hoping that this may influence the susceptibility of the β-ketoenol to bind metal ions.

**Scheme 1:**
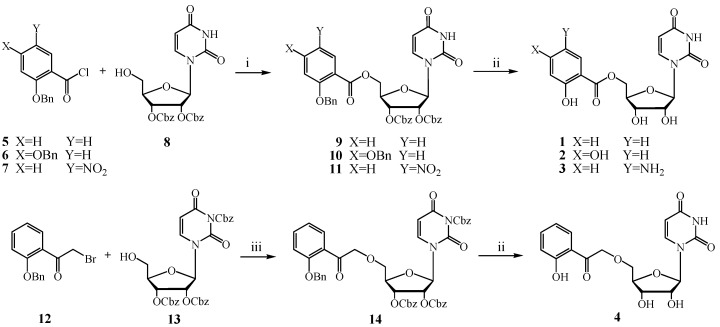
Synthesis of uridine derivatives **1**–**4**.

Ester derivatives **1**–**3** were easily synthesized by the reaction of 2′,3′-*O*-bis-(benzyloxycarbonyl) uridine (**8**) with acyl chlorides **5**–**7** (Scheme 1). Benzyloxycarbonyl groups were used for the protection of two secondary hydroxyl groups of uridine. Hydroxyl groups in the aromatic ring of aryl acyl chlorides were protected with the aid of benzyl groups. The main advantage of using benzyloxycarbonyl and benzyl protective groups is that they can be cleaved simultaneously by catalytic hydrogenolysis in the final step. Acyl chlorides **5**–**7** were synthesized in three-step syntheses starting from the corresponding methyl esters, which were benzylated, then hydrolyzed to acids and finally transformed to acyl chlorides using oxalyl chloride/DMF procedures [16,17,18]. Acylation reactions of uridine derivative **8** with crude acid chlorides **5**–**7** proceeded smoothly in the presence of pyridine in dry CH_2_Cl_2_ as a solvent. Under these conditions no acylation of the uracil nitrogen was observed. Totally deprotected compounds were obtained by catalytic hydrogenation with the use of cyclohexene as hydrogen donor [19] and Pd(OH)_2_/C as a catalyst [20]. We believed that the introduction of the electron withdrawing nitro group at the *para* position to the hydroxyl group in the benzene ring thus enhancing the acidicity of the phenol might lead to better coordination with metals. Therefore acyl chloride **7** possessing a nitro group was used, but unfortunately during hydrogenolysis the amino derivative **3** was formed as a main product.

*Reagents and Conditions:* (i) pyridine (10 equiv.), CH_2_Cl_2_, r.t., 24 h; (ii): 20% Pd(OH)_2_/C, cyclohexene, MeOH/THF, reflux, 1h; (iii): Cs_2_CO_3_ (6 equiv.), DMF, r.t., 2 h.

Formation of the ether derivative **4** according to the Williamson type synthesis *via* the alkoxide of the 5′-OH uridine derivative generated *in situ* by a NaH/THF system occurred in poor yield. It was observed that when uridine derivative **8** was used alkylation occurred preferably on the *N*-imide function of the uridine part and the reaction resulted in the formation of 3-alkylated uridine together with the 5′,N^3^-dialkylated uridine derivative. Therefore N^3^,2′,3′-O-tris-(benzyloxycarbonyl)uridine (**13**) was used as a substrate (Scheme 1). Due to the relatively low reactivity of both bromide **12** and uridine **13** the effect of activation with various bases, e.g., NaOH, NaH, CsOH, Ag_2_O, Ag_2_CO_3_, K_2_CO_3_, Cs_2_CO_3_ was studied. Finally the desired ether **4** was synthesized in reasonable yield by Cs_2_CO_3_-promoted O-alkylation [21]. All new compounds **1**–**4** were purified by column chromatography and their structures were elucidated with the aid of ^1^H and ^13^C-NMR spectroscopy data (including two-dimensional DQCOSY, HMQC, HMBC experiments and simulation analysis) and mass spectrometry analysis (for details see Experimental section).

The important part of this work was the biological evaluation of the synthesized compounds **1**–**4**. Firstly bovine milk β4GalT I was chosen as the target enzyme. It is one of the most well-studied enzymes among the commercially available glycosyltransferases. We have applied a fluorescence assay developed by Praly and co-workers [9]. This method was already applied for IC_50_ determination with another set of complex uridine derivatives in our previous study [22]. Compounds **1**–**4** were tested as potential inhibitors in a competition assay against β4GalT I using fluorescent acceptor β-GlcNAc-O-(CH_2_)_6_-dansyl as a substrate. None of the compounds presented in Figure 2 displayed any significant inhibitory activity against bovine milk β4GalT I at concentrations up to 2.0 mM.

The other valuable targets belonging to GT family are chitin synthase and 1,3-β-glucan synthase, which are involved in the biosynthesis of structural components of the cell wall of fungi [23,24]. Inhibition of these synthases can lead to development of new antifungal agents. The uridinyl moiety is present in natural peptidyl inhibitors of chitin synthase, e.g., nikkomycin Z and polyoxin D. Structural modifications of these natural compounds have been reported and several derivatives were identified as potent chitin synthase inhibitors with activity comparable to that of nikkomycin Z [11,25,26,27,28]. For example, Obi *et al*. [25] have synthesized compounds having hydrophobic aromatic groups connected through the amino acid part with uridine as very strong inhibitors of chitin synthase. Strong antifungal activity with simultaneous anti-chitin synthase activity was presented by Desphande *et al*. [28], who synthesized 1,2,3-triazoyl-linked uridine derivatives. These results prompted us to evaluate the synthesized compounds **1**–**4** for antifungal properties against *Aspergillus fumigatus* and *Candida albicans.* Experiments aiming at determination of MIC values of the synthesized compounds were carried out according to the EUCAST microdilution method [29,30]. Unfortunately no antifungal activity up to the maximum tested concentration of 1,000 µg/mL was observed for compounds **1**–**4**. Stock solutions of the tested compounds were prepared in DMSO due to their low water solubility and it was difficult to evaluate higher concentrations because of the interfering effect of DMSO.

The lack of antifungal activity observed for the tested uridine derivatives **1**–**4** does not preclude their inhibitory activity to fungal GTs, such as chitin or glucan synthases. The inhibition of these membrane proteins by any compound is strongly related to its ability to cross the cell wall and to achieve the targeted cell-structures, as well as to its resistance to enzymatic machinery of the cell, and even if a compound meets the above mentioned conditions it may be expelled from the cells by efflux without causing any disorders in the target. Anyway, the inactivity of the newly developed uridine derivatives against *Candida* and *Aspergillus* strains does not exclude that they may be active towards other fungal species. However, further testing as antifungal agents should be treated with reserve. Instead biological assays against fungal specific GTs should be carried out in order to confirm or negate enzymatic activity. The lack of antifungal activity excludes any compound as a candidate for an antifungal therapeutic, but does not cross out its usefulness as a biological tool, e.g., for the study of biological pathways and protein-ligand interactions. Biological assays of the synthesized compounds **1**–**4** towards chitin synthase are planned.

## 3. Experimental

### 3.1. General

Optical rotations were measured with a JASCO P-2000 polarimeter using a sodium lamp (589 nm) at room temperature. NMR spectra were recorded with a Varian spectrometer at a frequency of 300 MHz or 600 MHz using TMS as internal standard. Mass spectra were recorded with a WATERS LCT Premier XE system (high resolution mass spectrometer with TOF analyzer) using electrospray-ionization (ESI) technique. Reactions were monitored by TLC on precoated plates of silica gel 60 F254 (Merck) and visualized using UV light (254 nm). Crude products were purified using column chromatography performed on silica gel 60 (70–230 mesh, Fluka). Hexane/EtOAc or CHCl_3_/MeOH were used as solvent systems. All evaporations were performed under diminished pressure at 50 °C. Reversed phase HPLC analyses were performed using JASCO LC 2000 apparatus equipped with a reverse phase column (Nucleosil 100 C18, 5 µm, 25 × 0.4 cm; mobile phase: H_2_O/MeCN 73:27, flow rate 1 mL/min) with a fluorescence detector. Fluorescence of acceptor substrate and product was read at 385 nm excitation/540 nm emission.

2-(Benzyloxy)benzoyl chloride (**5**) [16], 2,4-bis(benzyloxy)benzoyl chloride (**6**) [17], 2-(benzyloxy)-5-nitrobenzoyl chloride (**7**) [18], 2′-benzyloxy-2-bromoacetophenone (**12**) [31], 2′,3′-*O*-bis-(benzyloxycarbonyl)uridine (**8**) [32] and N^3^,2′,3′-*O*-tris-(benzyloxycarbonyl) uridine (**13**) [33] were prepared according to the respective published procedures. Uridine 5′-diphophogalactose disodium salt (UDP-Gal), Pearlman catalyst 20% Pd(OH)_2_/C (50% wet) and other chemicals were purchased from Aldrich and Fluka and were used without purification. Bovine milk β-1,4-galactosyltransferase I (β4GalT I, 1 U/mg) was purchased from Sigma.

For the antifungal evaluation, *Candida albicans* ATCC 90028 (ATTC American Type Culture Collection) and *Aspergillus fumigatus* BCCM/IHEM 13934 (BCCM/IHEM Belgian Co-ordinated Collection of Microorganisms/Biomedical Fungi and Yeast Collection) were used. RPMI-1640 medium and MOPS (3-(*N*-morpholino)propanesulfonic acid) were purchased from Sigma.

### 3.2. Chemistry

#### 3.2.1. General Procedure for the Synthesis of Ester C-5′-Substituted Derivatives **1–3**

To a solution of 2′,3′-O-bis-(benzyloxycarbonyl)uridine (**8**, 0.5 mmol, 0.256 g) and pyridine (10 equiv., 0.41 mL) in dry CH_2_Cl_2_ (10 mL) crude acyl chloride **5**, **6** or **7** (2 equiv.) was added. The mixture was stirred at room temperature for 24 h. Then the mixture was diluted with CH_2_Cl_2_ (30 mL) and washed with water (3 × 30 mL). The organic phase was dried (MgSO_4_) and concentrated. Crude products were purified by column chromatography with a hexane-EtOAc 10:1→1:1 solvent system to yield protected intermediate products **9 **(78%), **10** (35%) or **11** (51%), respectively. For final deprotection **9**–**11** were dissolved in MeOH/THF (20 mL, 4:1 v/v), then 20% Pd(OH)_2_/C (0.200 g) and cyclohexene (2 mL) were added and the mixture was heated for 30–60 min. Then the mixture was cooled, the catalyst was removed by filtration and the filtrate was concentrated. Crude products was purified by column chromatography with a CHCl_3_-MeOH 50:1→10:1 gradient solvent system to give final products **1**(0.127 g, 91%), **2**(0.053 g, 88%) or **3**(0.029 g, 36%) as yellowish foamy solids.

*5′-(2′′-Hydroxybenzoyl)uridine* (**1**): [α]^20^_D_ = −39.9° (MeOH, c = 0.93), ^1^H-NMR (CD_3_OD, 300 MHz) δ: 4.25–4.28 (m, 3H, H-4′, H-5′a, H-5′b), 4.58 (dd, 1H, *J* = 4.2, *J* = 12.4 Hz, H-3′), 4.70 (dd, 1H, *J* = 2.7, *J* = 12.4 Hz, H-2′), 5.56 (d, 1H, *J* = 8.1 Hz, H-5), 5.83 (d, 1H, *J* = 2.7 Hz, H-1′), 6.91–6.98 (m, 2H, H-3′′, H-5′′), 7.51 (ddd, 1H, *J* = 1.7, *J* = 7.3 Hz, H-4′′), 7.64 (d, 1H, *J* = 8.1 Hz, H-6), 7.86 (dd, 1H, *J* = 1.7, 7.8 Hz, H-6′′). ^13^C-NMR (CD_3_OD) δ: 65.21 (C-5′), 71.28 (C-3′), 75.15 (C-2′), 82.68 (C-4′), 92.31 (C-1′), 102.84 (C-5), 113.48 (C-1′′), 118.64, 120.54 (C-3′′, C-5′′), 131.06 (C-6′′), 137.19 (C-4′′), 142.45 (C-6), 152.15 (C-2), 162.83 (C-2′′), 166.06 (C-4), 170.93 (C=O). ESI-HRMS m/z: 365.0985 (Calcd for C_16_H_17_O_8_N_2_: 365.0980).

*5′-(2′′,4′′-Dihydroxybenzoyl)uridine* (**2**): [α]_20_^D^ = −82.60° (MeOH, c = 1.00); ^1^H-NMR (CD_3_OD, 300 MHz) δ: 4.22–4.27 (m, 3H, H-4′, H-5′a, H-5′b), 4.52 (dd, 1H, *J* = 4.2, *J* = 12.2 Hz, H-3′), 4.68 (dd, 1H, *J* = 2.7, *J* = 12.2 Hz, H-2′), 5.56 (d, 1H, *J* = 8.1 Hz, H-5), 5.83 (d, 1H, *J* = 2.7 Hz, H-1′), 6.32 (d, 1H, *J* = 2.2 Hz, H-3′′), 6.37 (dd, 1H, *J* = 2.2, *J* = 8.8 Hz, H-5′′), 7.64 (d, 1H, *J* = 8.1 Hz, H-6), 7.70 (d, 1H, *J* = 8.8 Hz, H-6′′). ^13^C-NMR (CD_3_OD) δ: 64.55 (C-5′), 71.17 (C-3′), 75.26 (C-2′), 82.87 (C-4′), 92.04 (C-1′), 102.82 (C-5), 103.71 (C-3′′), 105.23 (C-1′′), 109.40 (C-5′′), 132.68 (C-6′′), 142.28 (C-6), 152.17 (C-2), 165.20 (C-4′′), 166.11 (C-4, C-2′′), 170.87 (C=O). ESI-HRMS m/z: 381.0934 (Calcd for C_16_H_17_O_9_N_2_: 381.0929).

*5′-(5′′-Amino-2′′-hydroxybenzoyl)uridine* (**3**): ^1^H-NMR (CD_3_OD, 600 MHz) δ: 4.19–4.28 (m, 3H, H-4′, H-5′a, H-5′b), 4.51 (dd, 1H, *J* = 4.1, *J* = 12.3 Hz, H-3′), 4.71 (dd, 1H, *J* = 2.9, *J* = 12.3 Hz, H-2′), 5.58 (d, 1H, *J* = 8.2 Hz, H-5), 5.82 (d, 1H, *J* = 2.9 Hz, H-1′), 6.77 (d, 1H, *J* = 8.8 Hz, H-3′′), 6.99 (dd, 1H, *J* = 2.9, *J* = 8.8 Hz, H-4′′), 7.25 (d, 1H, *J* = 2.9 Hz, H-6′′), 7.61 (d, 1H, *J* = 8.2 Hz, H-6). ^13^C-NMR (CD_3_OD) δ: 64.96 (C-5′), 71.159 (C-3′), 75.03 (C-2′), 82.76 (C-4′), 92.27 (C-1′), 102.92 (C-5), 113.16 (C-1′′), 116.29 (C-6′′), 119.04 (C-3′′), 129.29 (C-4′′), 140.34 (C-5′′), 142.44 (C-6), 152.14 (C-2), 155.91 (C-2′′), 169.32 (C-4), 170.84 (C=O). ESI-HRMS m/z: 380.1099 (Calcd for C_16_H_18_O_8_N_3_: 380.1094).

#### 3.2.2. Procedure for the Synthesis of Ether C-5′-Substituted Derivative **4**

To a solution of N^3^,2′,3′-O-tris-(benzyloxycarbonyl)uridine (**13**, 1.00 mmol, 0.646 g) in DMF (15 mL) 2′-benzyloxy-2-bromoacetophenone (**12**, 1.5 equiv., 0.457 g) and Cs_2_CO_3_ (6 equiv., 1.950 g) were added. The mixture was stirred at room temperature for 2 h. Then it was diluted with toluene and filtrated to remove solid Cs_2_CO_3_. The filtrate was neutralized with Dowex 50WX4 and concentrated. Crude product was purified by column chromatography with a hexane-AcOEt 50:1→5:1 solvent system to yield **14** as light yellow foamy solid (0.295 g, 34%). For final deprotection **14** was dissolved in a MeOH/THF 4:1 solvent system (20 mL), 20% Pd(OH)_2_/C (0.236 g) and cyclohexene (3.0 mL) were added and the mixture was heated under reflux for 50 min. Then the mixture was cooled, the catalyst was removed by filtration and the filtrate was concentrated. Crude product was purified by column chromatography with CHCl_3_:MeOH 100:1→30:1 solvent system to yield *5′-((2-(2-hydroxyphenyl)-2-oxoethyl)uridine* (**4**) as a cream foamy solid (0.073 g, 57%). [α]^20^_D_: −3.3° (c = 1.00, MeOH); ^1^H-NMR (DMSO, 600 MHz) δ: 3.70 (dd, 1H, *J* = 3.2, *J* = 10.7 Hz, H-5′b), 3.78 (dd, 1H, *J* = 3.0, *J* = 10.7 Hz, H-5′a), 4.01 (ddd, 1H, *J* = 3.0, *J* = 3.1, *J* = 3.2 Hz, H-4′), 4.07 (m, 1H, H-3′), 4.14 (ddd, 1H, *J* = 5.1, *J* = 5.5, J = 5.6 Hz, H-2′), 4.89–4.93 (qAB, 2H, J = 18.0 Hz, CH_2_), 5.20 (br s, 1H, 3′-OH), 5.42 (d, 1H, *J* = 5.5 Hz, 2′-OH), 5.62 (dd, 1H, *J* = 2.3, *J* = 8.1 Hz, H-5), 5.84 (d, 1H, *J* = 5.6 Hz, H-1′), 6.95 (ddd, 1H, *J* = 1.1, *J* = 7.2, *J* = 8.1 Hz, H-5′′), 7.00 (dd, 1H, *J* = 1.1, *J* = 8.4 Hz, H-3′′), 7.50 (ddd, 1H, *J* = 1.8, *J* = 7.2, *J* = 8.3, H-4′′), 7.78 (dd, 1H, *J* = 1.8, *J* = 8.0 Hz, H-6′′), 8.06 (d, 1H, *J* = 8.1 Hz, H-6), 11.20 (s, 1H, OH), 11.30 (d, 1H, *J* = 2.3 Hz, NH). ^13^C-NMR (DMSO) δ: 70.85 (C-3′), 70.86 (C-5′), 73.86 (C-2′), 75.60 (C-6′), 83.61 (C-4′), 88.21 (C-1′), 102.27 (C-5), 117.92 (C-3′′), 119.71 (C-5′′), 120.72 (C-1′′), 130.10 (C-6′′), 135.96 (C-4′′), 141.55 (C-6), 151.25 (C-2), 159.85 (C-2′′), 163.56 (C-4), 199.62 (C=O). ESI-HRMS m/z: 401.0848 (Calcd for C_17_H_18_O_8_N_2_: 401.0961).

### 3.3. Biological Evaluation

#### 3.3.1. Bovine Milk β-1,4-Galactosyltransferase I Assay

β4GalT I activity was assayed using UDP-Gal as glycosyl donor and β-GlcNAc-O-(CH_2_)_6_-dansyl as glycosyl acceptor [9]. Assays were performed in a total volume of 100 µL. The reaction mixtures contained reagents in the following final concentrations: 50 mM Hepes buffer (pH 7.4), 10 mM MnCl_2_, 2.0 mg/mL BSA, 200 µM β-GlcNAc-*O*-(CH_2_)_6_-dansyl, 40 µM UDP-Gal and potential inhibitors **1**–**4** at a range of concentrations from 0 mM (control) to 2.0 mM. The enzymatic reactions were started by the addition of 0.2 mU β4GalT I and incubated at 30 °C for 14 min. Inactivation was quickly done by immersion of the reaction solutions for 2 min in a boiling water bath. The solutions were diluted with water (200 µL) and centrifuged for 10 min, and the supernatant was injected into RP-HPLC system. The percentage of inhibition was evaluated from the fluorescence intensity of the peaks referring to product (Galβ-1,4-GlcNAcβ-O-(CH_2_)_6_-dansyl). Detailed results of the enzymatic assays are given in the supplementary material.

#### 3.3.2. Antifungal Assays

Broth microdilution technique was performed following the guidelines of the EUCAST [29,30]. 24 h old cultures of *C. albicans* ATCC 90028 and 2–3 days old cultures of *A. fumigatus* BCCM/IHEM13934 were used to prepare a suspension in RPMI-1640 medium of density of 2–5 × 10^5^ CFU/mL. The cells number were controlled by quantitative culture on Sabouraud agar medium and by microscopy using Burker’s chamber.

Examined compounds **1**–**4** were dissolved in DMSO to a stock concentration of 10 mg/mL. RPMI-1640 medium buffered to pH 7.0 with MOPS at a final concentration of 165 mM was used as a buffer to perform a followed concentrations of each compounds: 2,000, 1,000, 500, 250, 125, 62.5, 31.25, 15.625 and 7.81 µg/mL. Assays were carried out in 96-wells microdilution plates with flat bottom. The 100 µL aliquots of each concentration were distributed to the three wells of microdilution plates and subsequently each well was inoculated with 100 µL previously prepared fungal culture (except negative controls). As a positive and negative controls the cultures without addition of tested compounds and medium without tested microorganism were used, respectively. The plates were incubated at 35 °C for 24–48 h. The tests were read visually (*Aspergillus*, *Candida*) and/or by measure the absorbance of the samples at 530 nm (*Candida*). The tests were repeated three times for each strain.

### 3.4. In Silico Target Identification

To identify potential target candidates of compound **1**–**4**, *in silico* target screening using PharmMapper Server a reverse pharmacophore mapping approach was performed using an in-house pharmacophore database (PharmTargetDB) [15]. Uploaded mol2 files of the test molecules were built in ChemBio3D Ultra12. Possible geometries of analyzed ligands **1**–**4** were found using AM1 quantum semiempirical method. The default mapping parameters were used; maximum generated conformation: 300.

## 4. Conclusion

Synthesized new derivatives of uridine **1**–**4** were designed as donor substrate analogues of GTs. The β-ketoenol motif was expected to imitate the diphosphate bridge in UDP at the active site of GTs by chelating the divalent metal ion. No activity against β4GalT suggests that the β-ketoenol motif connected with uridine is not sufficient to ensure binding at the active site. Anyway the lack of activity against β4GalT opens the possibility of selectivity when other GTs would be tested.

## Supplementary Materials

Supplementary materials can be accessed at: http://www.mdpi.com/1420-3049/18/7/8018/s1.
