# Supplementary material for: Synthesis and Preliminary Biological Evaluations of 5′-Substituted Derivatives of Uridine as Glycosyltransferase Inhibitors
Source: Molecules. 2013 Jul 8;18(7):8018–27. doi: 10.3390/molecules18078018 (PMC6270623; doi:10.3390/molecules18078018)
Supplement: Supplementary file 1 [file molecules-18-08018-s001.pdf]

Supplementary Materials

Bovine milk β-1,4-galactosyltransferase I assay results

RP HPLC analyses were performed using Perkin Elmer Series 200 apparatus equipped with a reverse phase column (Nucleosil 100 C18, 5 µm, 25 × 0.4 cm; mobile phase: H_2O/MeCN 73:27, flow rate 1 mL/min) with a fluorescence detector. Fluorescence of acceptor substrate and product was read at 385 nm excitation/540 nm emission.

**Compound**	**Sample label**	**Conc. [mM]**	**Product peak area**	**Product %**	**Product retention [min]**	**Substrate peak area**	**Substrate [%]**	**Substrate retention [min]**	**Product/ Substrate ratio**
1	1	0	4319	4.605	18.602	88882	94.775	22.368	20.57930
	2	0.125	3935	4.253	18.545	88584	95.747	22.258	22.51182
	3	0.25	4949	4.905	18.205	95947	95.095	21.907	19.38715
	4	0.5	4792	4.960	18.316	91822	95.040	22.080	19.16152
	5	1	4284	4.214	18.782	97369	95.786	22.583	22.72852
	6	2	3708	4.437	18.402	80256	96.042	22.388	21.64401
2	7	0	3762	5.154	20.327	69223	94.846	24.532	18.40058
	8	0.125	3966	5.303	20.048	70817	94.697	24.300	17.85603
	9	0.25	4139	5.296	20.332	74009	94.704	24.488	17.88089
	10	0.5	4399	5.921	20.228	69889	94.079	24.478	15.88747
	11	1	4287	5.726	20.222	70573	94.274	24.412	16.46209
	12	2	3801	4.982	20.268	72496	95.018	24.422	19.07288
3	13	0	3585	5.405	20.298	62737	94.595	24.408	17.49986
	14	0.125	3918	5.416	20.275	68417	94.584	24.392	17.46223
	15	0.25	3902	5.447	20.185	67734	94.553	24.310	17.35879
	16	0.5	3852	5.424	20.173	67164	94.576	24.332	17.43614
	17	1	4290	5.518	20.638	73451	94.482	24.901	17.12145
	18	2	4354	5.491	20.838	74938	94.509	25.065	17.21130
4	19	0	3748	5.237	20.667	67827	94.763	24.887	18.09685
	20	0.125	3647	5.148	20.845	67202	94.852	25.093	18.42665
	21	0.25	4168	5.817	20.883	67474	94.183	25.045	16.18858
	22	0.5	4000	5.212	20.803	72744	94.788	24.983	18.18600
	23	1	4338	5.800	20.667	70460	94.200	24.850	16.24251
	24	2	3898	5.214	20.652	70480	94.282	24.828	18.08107

Average results: β-LacNAc-O-$(CH_2)_6$-dansyl (product): peak area 4064 ± 8%, retention time: 20 min, β-GlcNAc-O-$(CH_2)_6$-dansyl (acceptor substrate): peak area 75378 ± 11%, retention time: 24 min, Ratio of substrate/product: 18.56 ± 10%.
